# Low Prevalence and Abundance of *Akkermansia* in Older Japanese Adults: Inverse Association with a Noodle-Based Dietary Pattern but Not with Physical Function

**DOI:** 10.3390/microorganisms14081729

**Published:** 2026-08-06

**Authors:** Yuji Naito, Hiroaki Kitae, Katsura Mizushima, Norihiro Ouchi, Atsuo Adachi, Tadaaki Kamitani, Jin Narumoto, Tomoya Kitani, Satoaki Matoba, Ryo Inoue, Tomohisa Takagi

**Affiliations:** 1 Department of Human Immunology and Nutrition Science, Graduate School of Medical Science, Kyoto Prefectural University of Medicine, Kyoto 602-8566, Japan; hkitae@koto.kpu-m.ac.jp (H.K.);; 2 Molecular Gastroenterology and Hepatology, Graduate School of Medical Science, Kyoto Prefectural University of Medicine, Kyoto 602-8566, Japan; takatomo@koto.kpu-m.ac.jp; 3 Department of Internal Medicine, Kyotango Municipal Yasaka Hospital, Kyoto 627-0111, Japan; 4 Department of Psychiatry, Graduate School of Medical Science, Kyoto Prefectural University of Medicine, Kyoto 602-8566, Japan; jnaru@koto.kpu-m.ac.jp; 5 Department of Cardiovascular Medicine, Graduate School of Medical Science, Kyoto Prefectural University of Medicine, Kyoto 602-8566, Japan; t-kitani@koto.kpu-m.ac.jp (T.K.); matoba@koto.kpu-m.ac.jp (S.M.); 6 Department of Longevity and Regional Epidemiology, Graduate School of Medical Science, Kyoto Prefectural University of Medicine, Kyoto 602-8566, Japan; 7 Laboratory of Animal Science, Department of Applied Biological Sciences, Faculty of Agriculture, Setsunan University, Osaka 573-0101, Japan; ryo.inoue@setsunan.ac.jp

**Keywords:** *Akkermansia*, dietary pattern, principal component analysis, gut microbiota, noodles, grip strength, muscle mass, sarcopenia, frailty, older adults, compositional data analysis

## Abstract

*Akkermansia muciniphila* is regarded as a beneficial mucin-degrading commensal, yet in Japanese populations the genus *Akkermansia* occurs at low abundance and is frequently undetectable. We examined its dietary correlates and its relationship with physical function in 786 community-dwelling older Japanese adults (age ≥ 65 years). Dietary patterns were derived from 31 food groups by principal component analysis (PCA) with varimax rotation, and *Akkermansia* was quantified by 16S rRNA gene sequencing and expressed as centered log-ratio (CLR)-transformed z-scores. *Akkermansia* was undetectable in 356 participants (45.3%) and of low abundance among carriers (median ≈ 0.35%). A noodle-based dietary pattern (principal component [PC] 4) was inversely associated with *Akkermansia* after adjustment for age, sex, body mass index, and lifestyle/comorbidity covariates (β = −0.125; *p* = 0.0007), with a dose–response across quartiles (*p* for trend = 0.004). In a two-part model, this association was confined to detection (adjusted odds ratio per 1 standard deviation = 0.80; 95% confidence interval, 0.69–0.93) and was absent among carriers (*p* = 0.25), indicating that diet was related to whether *Akkermansia* was present rather than to its abundance. *Akkermansia* was unrelated to grip strength, muscle mass, and frailty; weak carrier-restricted associations did not survive false discovery rate correction (*p* = 0.056) and are considered exploratory. These findings provide limited evidence for a role of *Akkermansia* in physical function in this low-abundance population.

## 1. Introduction

*Akkermansia muciniphila*, a mucin-degrading bacterium residing in the intestinal mucus layer, has emerged as a leading next-generation probiotic [1,2]. Its abundance is inversely correlated with body mass index (BMI) and fasting glucose levels, and it strengthens gut barrier integrity, modulates mucosal immunity, and ameliorates features of obesity and type 2 diabetes [3,4,5,6]. A human proof-of-concept trial has reported improved insulin sensitivity after supplementation with pasteurized *A. muciniphila* [7], and higher abundance of this species is associated with favorable responses to programmed cell death protein 1-based cancer immunotherapy [8] and neuroprotection in an amyotrophic lateral sclerosis model [9]. However, its effects are increasingly recognized as context-dependent. Under low-fiber conditions or altered microbial ecology, excessive mucin degradation may compromise the mucus barrier, and *A. muciniphila* enrichment is associated with adverse outcomes in some inflammatory and oncologic models [10,11,12]. These observations suggest that a higher relative abundance of *A. muciniphila* is not invariably beneficial. Notably, this evidence base derives almost entirely from Western populations and experimental models, and its relevance to Japanese populations—in whom the genus is uncommon—has not been established.

Dietary factors are among the most tractable modulators of *A. muciniphila*. The intake of dietary fiber and polyphenols, particularly proanthocyanidins, can promote its growth, although its effects depend on the broader dietary context [13,14,15]. Because foods are consumed in combination, dietary pattern approaches, typically principal component analysis (PCA) with varimax rotation to derive interpretable patterns, are well established in nutritional epidemiology and capture diet–microbiome relationships better than single nutrients [16,17]. Beyond fiber, other dietary components—including artificial sweeteners, emulsifiers, and other processed-food constituents—are increasingly recognized as modulators of gut microbial composition and represent potential dietary confounders [18], and diet–microbiome interactions are of broad relevance to intestinal health [19].

Importantly, the gut microbiome of Japanese individuals differs markedly from that of Western populations, with distinctive enrichment of certain taxa, generally low *Akkermansia* abundance, and a reported sex difference toward higher abundance in women [20,21,22,23]. In such low-abundance zero-inflated settings, the analytical distinction between presence (detection) and quantity (abundance among carriers) is critical and best handled using compositional methods and two-part models [24].

Simultaneously, the gut–muscle and gut–aging axes have attracted attention. *Akkermansia* is enriched in centenarians, and its restoration extends the healthspan and lifespan of progeroid mice [25]. Preclinical and small clinical studies have suggested that *A. muciniphila* protects against sarcopenia and improves muscle strength, and patients with sarcopenia exhibit low *Akkermansia* abundance [26,27,28]. Gut dysbiosis is associated with frailty [29,30]. However, whether these largely Western interventional findings can be generalized to low-*Akkermansia*-abundance Japanese populations remains unknown.

In 786 community-dwelling older Japanese adults, we (i) identified the dietary patterns associated with *Akkermansia* abundance and (ii) investigated whether *Akkermansia* relates to grip strength, muscle mass, and frailty, explicitly separating detection from abundance using a two-part modeling framework.

## 2. Materials and Methods

### 2.1. Study Population

Between 1 January 2017 and 31 December 2021, 786 participants (age ≥ 65 years) provided fecal samples and completed dietary and lifestyle questionnaires. This study adhered to the Declaration of Helsinki and was approved by the Ethics Committee of Kyoto Prefectural University of Medicine (approval number: ERB-C-885-9). All participants provided written informed consent before enrollment. This study was registered in the University Hospital Medical Information Network Clinical Trials Registry (UMIN000019486) on 1 December 2015.

### 2.2. Dietary Assessment

Habitual dietary intake during the month preceding the study was assessed using the Brief-type Self-administered Diet History Questionnaire, a validated 70-item food-frequency instrument widely used in Japanese epidemiological studies [31,32]. Although this questionnaire is designed for self-completion, trained public health nurses (hokenshi) at the survey sites supported each participant to maximize response accuracy and minimize missing data among the older adult population, as all participants were aged ≥65 years. Support included clarifying item wording, reviewing recall of portion sizes and frequencies, and verifying that all items had been answered. The estimated intake of 70 food items was adjusted for total energy intake using the density method (per 1000 kcal) and standardized to z-scores. To improve the interpretability and stability of pattern extraction, all items were aggregated into 31 food groups based on their nutritional and culinary similarities (Supplementary Table S1). Three seasonal duplicate items (citrus, persimmon, and strawberry) were removed.

### 2.3. Analysis of Fecal Microbiota

Quality-filtered sequencing depths ranged from 21,105 to 775,188 reads per sample (median, 84,465 reads) among the 786 samples. Because dependable detection (≥95% probability) of a taxon present at approximately 0.005% relative abundance would require on the order of 60,000 reads—a depth not reached in all samples—and because a small number of spurious reads can arise from index (barcode) hopping, we did not adopt a single fixed relative-abundance detection limit. *Akkermansia* was classified as undetectable when no *Akkermansia*-assigned reads were observed, and a conservative 0.25% relative-abundance threshold was additionally applied as a sensitivity analysis [33].

Fecal microbial DNA was extracted using the Maxwell RSC Fecal Microbiome DNA Kit (Promega, Tokyo, Japan), according to the manufacturer’s instructions. The V3–V4 region of 16S rRNA was amplified using the primers 341F (5′-CCTACGGGNGGCWGCAG-3′) and 805R (5′-GACTACHVGGGTATCTAATCC-3′) and the Tks Gflex DNA Polymerase (TaKaRa Bio, Kusatsu, Japan). Polymerase chain reaction (PCR) amplification was performed using the following thermal cycling conditions: An initial denaturation at 95 °C for 3 min, followed by 25 cycles of denaturation at 95 °C for 30 s, annealing at 55 °C for 30 s, and extension at 72 °C for 30 s, concluding with a final elongation step at 72 °C for 5 min. The resulting amplicons were purified using the NucleoFast96 PCR Plates (TaKaRa Bio), and indexing PCR was subsequently performed using unique dual-index primer sets compatible with MiSeq sequencing (Illumina, San Diego, CA, USA), according to the manufacturer’s protocol. After indexing, the PCR products were purified, normalized using the SequalPrep Normalization Plate Kit (Life Technologies, Tokyo, Japan), and pooled at equimolar concentrations. The pooled library was purified using AMPure XP magnetic beads (Beckman Coulter, Brea, CA, USA). The resulting purified library was subjected to 285 bp paired-end sequencing on the Illumina MiSeq platform using the MiSeq Reagent Kit v3. The MiSeq sequencing data were processed using QIIME 2 version 2024.5 [34,35]. Demultiplexed paired-end reads were imported in the PairedEndFastqManifestPhred33 format. Quality filtering, paired-end read merging, chimera removal, and amplicon sequence variant (ASV) inference were performed using the q2-dada2 plugin (dada2 denoise-paired). The first 17 and 19 bases were trimmed from the forward and reverse reads, respectively, and the forward and reverse reads were truncated at 260 and 240 bases, respectively. Taxonomy was assigned using the q2-feature-classifier plugin (feature-classifier classify-sklearn) with a SILVA 138 reference classifier clustered at 99% sequence identity [36]. ASVs with a total frequency of fewer than two reads across the dataset were removed using q2-feature-table (feature-table filter-features, minimum frequency = 2). Representative ASV sequences were phylogenetically placed using q2-fragment-insertion (fragment-insertion sepp) with the SILVA 128 SEPP reference database, and ASVs that could not be placed in the reference tree were excluded. Features classified as mitochondria or chloroplasts were removed using q2-taxa (taxa filter-table), and only features containing a phylum-level taxonomic assignment were retained. The filtered feature table was collapsed at the genus level using q2-taxa (taxa collapse, level 6), and relative abundances were calculated using q2-feature-table (feature-table relative-frequency). Unless otherwise specified, default parameters in QIIME 2 version 2024.5 were used. The relative abundance data of *Akkermansia* were transformed using a centered log-ratio (CLR) transformation [24] to account for compositionality; subsequent analyses were conducted using the CLR-transformed and z-standardized genus-level data. Before a CLR transformation, zero values were replaced by adding a pseudocount of [1.0 × 10^−6^] to all genus-level abundances.

The raw 16S rRNA gene sequencing data generated in this study have been deposited in the National Center for Biotechnology Information Sequence Read Archive under accession numbers PRJNA1470357 and PRJNA1440982, and the corresponding subject-level identifier list (Microbiota-ID, age, sex, and BMI) is provided in Supplementary Table S2. All other relevant data supporting the findings of this study are included in the article and its Supplementary Material files.

To resolve *Akkermansia* to the species level, all ASVs classified by SILVA as *Akkermansia* or *Akkermansiaceae* were compared with reference V3–V4 amplicons of validly named *Akkermansia* species retrieved from GenBank. Reference 16S rRNA gene sequences for the *Akkermansia* species listed in the List of Prokaryotic names with Standing in Nomenclature (LPSN) were retrieved from GenBank (accessions LC711105, KT254068, DXEH01000042, ON014381, OP143661, and CP072051). Sequences were oriented as necessary, and the regions between the 341F and 805R primer-binding sites were extracted in silico with the primer sequences excluded. The resulting reference sequences were aligned with all ASVs classified by SILVA as *Akkermansia* or *Akkermansiaceae* using MAFFT. Pairwise nucleotide identity was calculated over aligned, unambiguous nucleotide positions. Because strain-level discrimination was not attempted, an ASV was assigned at the species level when it showed at least 98% nucleotide identity and a unique best match to a reference species. ASVs with co-best matches to multiple species were regarded as an unresolved species group, and ASVs with less than 98% identity were retained as *Akkermansia* sp.

### 2.4. Physical Function and Covariates

Frailty was operationalized using the deficit-accumulation framework of Searle and colleagues [37]. From a 40-item candidate deficit list—covering self-reported symptoms, signs, anthropometric measures, laboratory abnormalities, comorbidities and functional indicators—we retained the 32 items that could be unambiguously coded from the Kyotango Cohort data set, comprising clinical and biochemical measurements, anthropometry, lifestyle and medical-history questionnaire items, and self-rated functional status. The frailty index was calculated as the proportion of deficits present (range 0–1), with participants missing more than 20% of items excluded from the index [38]. Grip strength, skeletal muscle mass, and the frailty index were expressed as z-scores. Covariates comprised age, sex, BMI, and lifestyle/comorbidity factors (smoking, alcohol consumption, constipation, diabetes, hypertension, polypharmacy, and exercise habits).

### 2.5. Statistical Analyses

Dietary patterns were derived via PCA of the 31 standardized food groups, followed by varimax rotation [16,17]. Eleven components (eigenvalue > 1; cumulative variance, 61.1%) were retained and used to compute standardized regression-based component scores. Participants were classified using a hybrid procedure: Ward’s hierarchical clustering of the retained rotated component scores provided initial centroids that were refined by k-means, and the number of clusters was selected in a data-driven manner based on the silhouette criterion. Associations between component scores and *Akkermansia* CLR-Z were determined using linear regression with Benjamini–Hochberg false discovery rate (FDR) correction, and between-cluster differences were analyzed using the Kruskal–Wallis test. The leading pattern (PC4) was modeled as crude and with progressive adjustment (core: Age, sex, and BMI; extended: Core + lifestyle/comorbidity), complemented by a quartile trend test and logistic model for detection. For physical function, a two-part model was applied: (1) Logistic regression of *Akkermansia* detection on each phenotype and (2) linear regression of each phenotype on *Akkermansia* CLR-Z restricted to carriers, both adjusted for age, sex, and BMI. All analyses were conducted using Python 3.11 (statsmodels, scikit-learn, and SciPy), and a two-sided *p* < 0.05 was considered significant. The diet–Akkermansia association was additionally examined within a two-part (hurdle) framework comprising logistic regression for detection and linear regression restricted to carriers. For the physical-function analyses, Benjamini–Hochberg FDR correction was applied across all nine models (three phenotypes × three analytical approaches).

## 3. Results

### 3.1. Participants and Akkermansia Distribution

**Figure 1:**
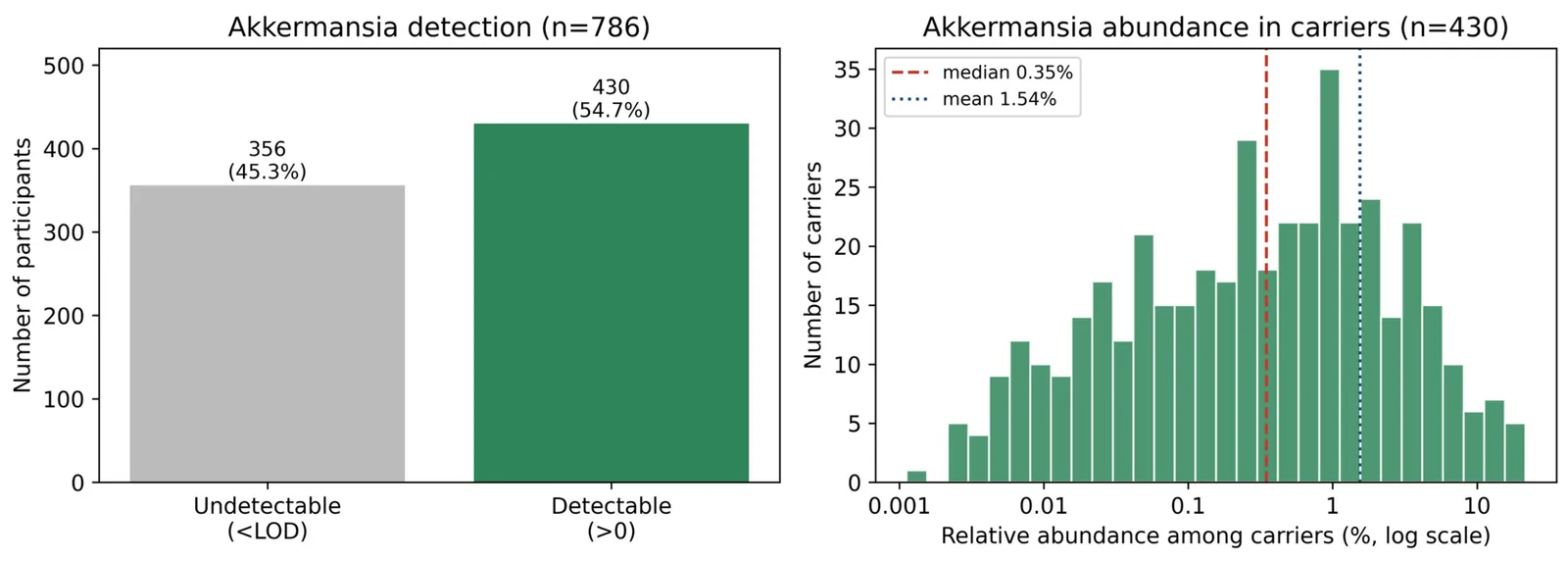
Distribution of *Akkermansia* abundance in the study cohort. **Left**: Number of participants with undetectable (relative abundance = 0; below the limit of detection [LOD]) versus detectable (>0) *Akkermansia* (*n* = 786). **Right**: Histogram of relative abundance among carriers (*n* = 430) on a log scale. The dashed line indicates the median (0.35%), whereas the dotted line indicates the mean (1.54%). *Akkermansia* was undetectable in 356 participants (45.3%), and its abundance was low even among carriers (maximum 21.6%), confirming a low-abundance zero-inflated distribution. LOD, limit of detection.

*Akkermansia* was undetectable (no assigned reads) in 356 of the 786 participants (45.3%) and detectable in 430 (54.7%); among carriers, the relative abundance was low (median 0.35%, maximum 21.6%), confirming a low-abundance, zero-inflated distribution (Figure 1). Because reliable detection of a taxon at approximately 0.005% relative abundance would require on the order of 60,000 reads, a depth not reached in all samples, and because low-level reads can arise from index (barcode) hopping, we did not treat 0.005% as a formal detection limit; instead, detection was defined as the presence of any *Akkermansia*-assigned read, and a conservative 0.25% relative-abundance threshold was additionally applied as a sensitivity analysis [33] (Supplementary Table S10).

Using these above-mentioned criteria, 25 of the 52 detected ASVs were assigned to a single species. These ASVs accounted for 644,447 of 660,880 *Akkermansia* reads (97.51%): 20 ASVs were assigned to *A. muciniphila* (533,791 reads; 80.77%) and five to *A. massiliensis* (110,656 reads; 16.74%). Three ASVs accounting for 16,273 reads (2.46%) matched *A. biwaensis* and “*Candidatus* A. timonensis” equally and were therefore retained as an unresolved species group. The remaining 24 ASVs were below the 98% threshold but together accounted for only 160 reads (0.024%). Species-level reanalysis indicated that *A. muciniphila* was the predominant species, accounting for more than 80% of *Akkermansia* reads (80.77%). *A. massiliensis* accounted for 16.74%, whereas 2.46% of reads could not be distinguished between *A. biwaensis* and “*Candidatus* A. timonensis.” ASVs below the 98% assignment threshold accounted for only 0.024% of reads (Supplementary Table S9). The genus in this cohort therefore comprised predominantly *A. muciniphila*, with a substantial minority of *A. massiliensis*.

### 3.2. Dietary Patterns

PCA with varimax rotation of the 31 food groups yielded interpretable patterns. The loading structure of the first five components is shown in Supplementary Figure S1. The component associated with *Akkermansia*, PC4, contrasted a noodle-and-convenience-food pattern with a rice-and-vegetable pattern: It loaded positively on noodles (0.84), salt/soy seasonings including noodle broth (0.79), fried foods (0.30), pickles (0.25), and alcohol (0.24) and negatively on rice (−0.32), green-and-yellow vegetables (−0.16), and fruits (−0.15). Therefore, a higher PC4 score denotes a more noodle-and-convenience-food-oriented diet with less rice, vegetables, and fruits (Supplementary Figure S1). A data-driven cluster analysis was concordant but weak and is reported in the Supplementary Materials (Supplementary Figure S3; Supplementary Table S5). The two-cluster solution exhibited a low silhouette (approximately 0.05) and separated participants mainly along a component unrelated to *Akkermansia* (PC8); therefore, inference was based on the continuous component scores.

### 3.3. Dietary Patterns and Akkermansia Abundance

**Figure 2:**
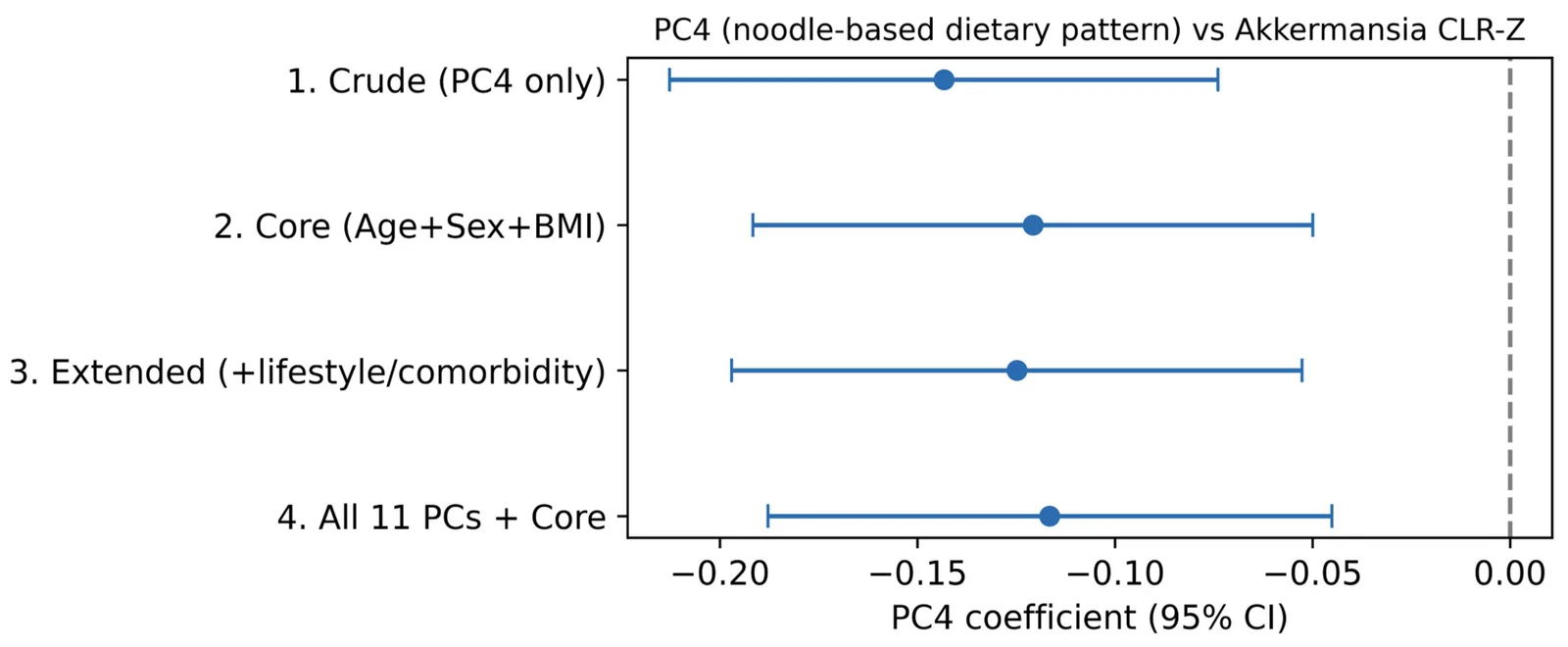
Association between principal component (PC)-4 and *Akkermansia* abundance across regression models. Points show the regression coefficient (β) for PC4 predicting centered log-ratio (CLR)-transformed Z-standardized *Akkermansia* abundance; horizontal bars represent 95% confidence intervals. A higher PC4 score corresponds to greater noodle (and convenience food) intake; negative β indicates that greater noodle intake is associated with lower *Akkermansia* abundance. Models: (1) Crude (PC4 only); (2) core, adjusted for age, sex, and body mass index (BMI); (3) extended, additionally adjusted for smoking, alcohol consumption, constipation, diabetes, hypertension, polypharmacy, and exercise habits; and (4) all 11 retained components entered simultaneously together with the core covariates (*n* = 786). The 95% confidence interval excludes the null (dashed line; β = 0) in every model.

Of the 11 components, only PC4 was significantly associated with *Akkermansia* CLR-Z (crude β = −0.143, *p* = 0.0001; FDR-significant, FDR-adjusted *p* = 0.0095). Notably, the inverse association persisted after core and extended adjustments (Table 1; Figure 2).

To identify the food groups driving this association, we regressed *Akkermansia* CLR-Z on each of the 31 food groups (adjusted for age, sex, and BMI; Supplementary Table S3). Inverse associations were concentrated in noodle-related and convenience groups, i.e., noodles (β = −0.080; *p* = 0.028), salt/soy seasonings including noodle broth (β = −0.082; *p* = 0.021), soy (β = −0.095; *p* = 0.008), and pickles (β = −0.084; *p* = 0.019), whereas rice (β = 0.021; *p* = 0.56), meat (β = 0.008; *p* = 0.83), and green-and-yellow vegetables (β = 0.001; *p* = 0.98) were not associated. Although no single group survived FDR correction, the pattern mirrored the PC4 loadings and indicated that the diet–*Akkermansia* relationship was driven by greater noodle and convenience food intake rather than rice, meat, or vegetable intake.

PC4 also loaded on salt and soy seasonings, and dietary sodium and salt-equivalent intake showed nominal inverse associations with *Akkermansia* CLR-Z (β ≈ −0.086; *p* = 0.015), although neither survived FDR correction. Within this group, the signal was carried by noodle broth (β = −0.085; *p* = 0.020), a marker of noodle consumption, whereas soy sauce and cooking salt were not independently associated, and noodle and salt/seasoning intakes were collinear and could not be statistically separated (both attenuated to non-significance in a mutually adjusted model). These findings suggest a high-salt refined-wheat convenience-eating style as a plausible correlate; however, the effects of dietary salt distinct from noodle consumption warrant further investigation.

Because specific medication classes may affect the gut microbiota, we additionally adjusted the primary model for six medication/comorbidity indicators recorded in the cohort: proton-pump inhibitors (*n* = 24, 3.1%), antibiotics (*n* = 5, 0.6%), statins (*n* = 212, 27.0%), antidiabetic treatment/diabetes (*n* = 75, 9.5%), laxatives (*n* = 59, 7.5%), and antihypertensive treatment/hypertension (*n* = 279, 35.5%). The association of PC4 with *Akkermansia* was unchanged (β = −0.122; 95% CI, −0.193 to −0.051; *p* = 0.0008). None of the six medications was individually associated with *Akkermansia* CLR-Z or with its detection (all *p* > 0.2; Supplementary Table S7), and none was associated with the noodle-based pattern (PC4; all *p* > 0.5), indicating that these medications did not confound the diet–*Akkermansia* association. Antibiotic use was very infrequent (0.6%), which limited power to assess its effect.

We further examined gastrointestinal, dietary, and social variables that could plausibly influence both noodle consumption and the gut microbiota. Additional adjustment for total energy intake, bowel frequency and stool consistency (Bristol Stool Scale), social support (Lubben Social Network Scale-6) and ikigai (sense of life worth), dental-visit frequency, and exercise habit left the PC4–*Akkermansia* association essentially unchanged, and a fully adjusted model including all of these variables yielded β = −0.127 (95% CI, −0.202 to −0.053; *p* = 0.0009; *n* = 752). None of these variables was individually associated with *Akkermansia* (all *p* > 0.14; Supplementary Table S8). Stool consistency and bowel frequency, in particular, did not attenuate the association, indicating that the diet–*Akkermansia* relationship was not explained by differences in bowel habit.

In contrast to a fiber-mediated pathway, neither total nor fractionated (soluble or insoluble) dietary fiber was associated with *Akkermansia* abundance (total fiber β = −0.027; *p* = 0.47) or its detection, and a quartile analysis of total fiber showed no trend (*p* = 0.63). Moreover, noodle intake was not correlated with lower fiber intake (β = −0.001; *p* = 0.99), and adjustment for total dietary fiber did not attenuate the noodle association with *Akkermansia* abundance (0% attenuation). None of the 96 nutrients survived FDR correction. Therefore, the noodle–*Akkermansia* relationship was independent of measured dietary fiber and other nutrients (Supplementary Table S4).

**Figure 3:**
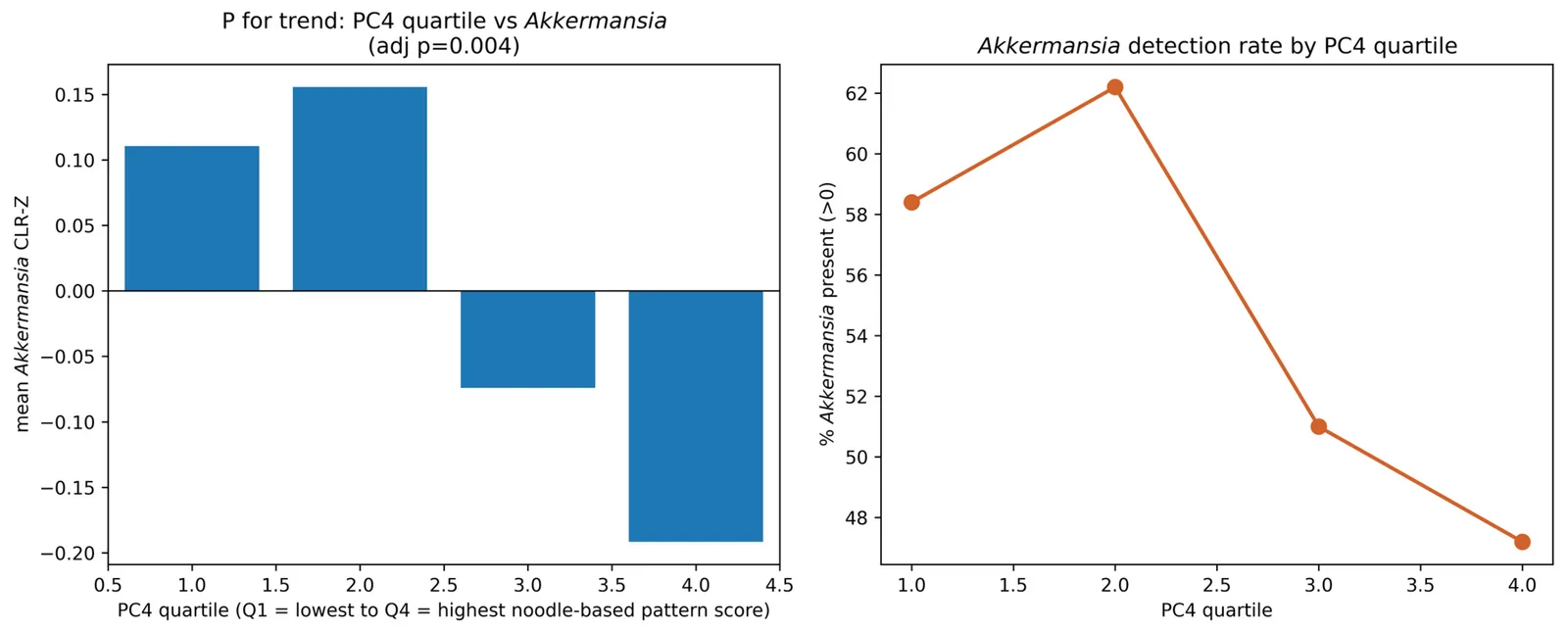
Dose–response relationship between PC4 and *Akkermansia* across quartiles. The participants (*n* = 786) were divided into quartiles of the PC4 score (Q1 = fewest noodles; Q4 = most noodles). Left panel: Mean CLR-transformed Z-standardized *Akkermansia* abundance by quartile. Right panel: Percentage of participants with detectable *Akkermansia* (relative abundance > 0%) by quartile. Both the mean abundance and detection rate decreased monotonically from Q1 to Q4 (linear trend across quartiles adjusted for age, sex, and BMI, *p* = 0.004; Kruskal–Wallis across quartiles, *p* = 0.0015).

Sensitivity analyses reinforced the above-mentioned findings. Across PC4 quartiles, mean *Akkermansia* CLR-Z decreased monotonically from Q1 (fewest noodles) to Q4 (most noodles; median: +0.31 → −0.63), and the detection rate fell from 58.4% to 47.2% (*p* for trend: crude 0.0004, adjusted 0.0043; Kruskal–Wallis across quartiles *p* = 0.0015; Figure 3). In logistic regression, each 1-standard deviation increase in PC4 (greater noodle intake) lowered the odds of *Akkermansia* detection (crude odds ratio [OR] = 0.78, 95% confidence interval [CI] = 0.67–0.90; adjusted OR = 0.80, 95% CI = 0.69–0.93; *p* = 0.003).

In a two-part (hurdle) sensitivity analysis, this association was confined to the detection component. Each 1-SD increase in PC4 lowered the adjusted odds of *Akkermansia* detection (OR = 0.80; 95% CI = 0.69–0.93; *p* = 0.003), whereas PC4 was not associated with abundance among carriers (adjusted β = −0.037; 95% CI = −0.100 to 0.026; *p* = 0.25). Thus, the diet–*Akkermansia* relationship reflected whether *Akkermansia* was detectable rather than its abundance among carriers. The association was also robust to the definition of detection: when a conservative 0.25% relative-abundance threshold was applied (reclassifying 236 participants [30.0%] as carriers), a higher PC4 score remained associated with lower odds of *Akkermansia* detection (adjusted OR per 1 SD = 0.78; 95% CI = 0.66–0.93; *p* = 0.005), with a monotonic decrease across PC4 quartiles (34.0% to 20.3%), whereas PC4 remained unassociated with abundance among these higher-confidence carriers (*p* = 0.92; Supplementary Table S10).

### 3.4. Akkermansia and Physical Function (Two-Part Model)

*Akkermansia* showed no meaningful relationship with physical function. In the full sample, weak crude inverse correlations of *Akkermansia* with grip strength and muscle mass (r ≈ −0.09; *p* < 0.02) were abolished after adjusting for age, sex, and BMI (grip *p* = 0.33; muscle *p* = 0.49), and frailty was unrelated throughout. Because of zero inflation, we additionally applied a two-part model. The detection of *Akkermansia* (presence vs. absence) was unrelated to grip strength or muscle mass (Table 2, Part 1), indicating that carrying the taxon was not associated with these phenotypes. In exploratory analyses restricted to carriers, higher *Akkermansia* CLR-Z was weakly associated with lower grip strength and muscle mass (Table 2, Part 2; Supplementary Figure S2), whereas frailty again showed no association (*p* > 0.9). However, after Benjamini–Hochberg correction across the nine physical-function models, neither carrier-restricted association remained statistically significant (FDR-adjusted *p* = 0.056 for both). Considering the very low abundances involved and the loss of significance after correction for multiple testing, these carrier-restricted signals are reported for completeness and interpreted as exploratory rather than as robust effects.

Finally, to determine whether another genus might occupy the functional niche attributed to *Akkermansia*, we screened all 147 genus-level taxa for associations with the three physical-function phenotypes and with the noodle-based pattern (PC4), adjusted for age, sex, and BMI and corrected using the Benjamini–Hochberg procedure (Supplementary Table S6; Supplementary Figure S5). No genus emerged as a functional substitute for physical function: across 441 taxon–phenotype tests, only *Megasphaera* was FDR-significantly (inversely) associated with grip strength (β = −0.135; *p* = 0.00003; FDR-adjusted *p* = 0.004), and no taxon was FDR-significant for muscle mass or frailty; *Akkermansia* ranked 65th, 100th, and 140th of 147 taxa for these phenotypes, respectively. For diet, *Akkermansia* ranked first of 147 taxa in its association with PC4 (β = −0.117; *p* = 0.0009), with *Oscillibacter* showing a comparable association in the opposite direction; across all taxa these corresponded to FDR-adjusted *p* = 0.064. This taxa-wide screen was exploratory, and *Akkermansia* being the top-ranked taxon supports the specificity of the reported diet–*Akkermansia* association.

## 4. Discussion

Although the primary analyses were performed at the genus level, the observed *Akkermansia* signal predominantly represented *A. muciniphila*. Nevertheless, because approximately 19% of *Akkermansia* reads were assigned to other species or unresolved species groups, the observed dietary association should not be interpreted as exclusively specific to *A. muciniphila*. The first observation was the rarity of *Akkermansia* in the studied population. Consistent with previous reports in Japanese cohorts [20,21,22], this genus was present below the detection limit in 45.3% of the participants, and its relative abundance was low even among carriers (mean ≈ 1.5%; median ≈ 0.35%), considerably lower than the values commonly reported in Western populations. This pronounced zero inflation and low carriage have both analytical and biological implications. Analytically, they mandated approaches that separate detection from abundance: a single linear model fitted to the whole sample masked the carrier-restricted associations with muscle phenotypes that became apparent only after applying the two-part model. Biologically, inferences regarding *Akkermansia* drawn from higher-abundance Western cohorts may not translate directly to Japanese populations, in which the ecological niche and host context of the taxon differ.

In this cohort of older Japanese adults, the dietary correlate of *Akkermansia* abundance was specifically noodle intake: Greater consumption of single-bowl noodle dishes, especially ramen and noodle-broth meals, was associated with lower *Akkermansia* abundance, whereas rice, meat, and vegetable intakes showed no independent associations. This inverse noodle-specific relationship was robust and dose-dependent across continuous regression, quartile-trend, and detection analyses and stable after adjustment for demographic, lifestyle, and comorbidity covariates. Although dietary fiber and polyphenols promote *A. muciniphila* growth in experimental and other observational settings [13,14,15], the association was unexpectedly independent of measured fiber in our cohort. Neither total nor fractionated dietary fiber was related to *Akkermansia* abundance, and fiber did not mediate the noodle–*Akkermansia* association. Therefore, the operative correlate was reduced noodle intake rather than a positive effect of a rice-and-side-dish (“washoku”) pattern or higher fiber intake. Although we anticipated a fiber-mediated pathway, the data did not support this. Accordingly, the mechanism by which higher noodle intake was associated with lower *Akkermansia* abundance could not be elucidated in this study. Whether factors such as glycemic load, food additives or emulsifiers, lower dietary diversity, or displacement of other *Akkermansia*-supporting foods are involved warrants further investigation.

*Akkermansia* showed no meaningful relationship with physical function. Its detection (presence vs. absence) was unrelated to grip strength, muscle mass, or frailty, and no association persisted in the full sample after adjusting for age, sex, and BMI. This null result is the more interpretable finding, as the genus was absent in nearly half of the participants and present at very low abundance in the remainder. In such a setting, the presence or absence of *Akkermansia* is unlikely to be a major determinant of muscle phenotypes in older Japanese adults. Weak inverse associations of abundance with grip strength and muscle mass were observed only in the exploratory carrier-restricted analyses. Notably, neither carrier-restricted association remained significant after correction for multiple testing across the nine physical-function models (FDR-adjusted *p* = 0.056), reinforcing their exploratory status. We refrain from emphasizing this signal as it rests on a conditional subgroup with extremely low abundances, the cross-sectional design precludes causal inference (reverse causation or residual confounding cannot be excluded), and it is not supported by detection-level analysis. Although some preclinical and small clinical studies have suggested that *A. muciniphila* benefits muscles and that patients with sarcopenia show low Akkermansia abundance [26,27,28], our data neither confirm nor contradict such effects in a population where the taxon is largely absent or minimally abundant.

Taken together, our findings support a diet–*Akkermansia* relationship (a lower noodle-based dietary pattern score associated with a higher likelihood of *Akkermansia* detection, independent of measured dietary fiber) and indicate that the presence or absence of *Akkermansia* had no clear impact on grip strength, muscle mass, or frailty in this low-*Akkermansia*-abundance Japanese population. The strengths of this study include a sizeable cohort, principled compositional treatment of a zero-inflated taxon, multi-method triangulation, and a two-part framework that separates detection from abundance. Its limitations include the cross-sectional design, self-reported diet, single-cohort generalizability, reliance on relative (compositional) rather than absolute abundance, the absence of adjustment for sequencing batch, the exploratory and conditional nature of carrier-restricted analyses, and modest explained variance (R^2^ ≈ 0.02–0.06), indicating that diet is one of several determinants of *Akkermansia* abundance. Notably, the bimodal presence/absence of *Akkermansia*, together with its near absence in Japanese infants and children, in whom enterotypes are dominated by Bifidobacterium, Bacteroides, or Klebsiella rather than *Akkermansia* [39,40], raises the possibility that host and early life factors, rather than adult diet, contribute substantially to *Akkermansia* colonization in Japanese populations. However, this hypothesis was not directly tested in the present study and therefore remains speculative and requires dedicated investigation. Species-level re-analysis of all *Akkermansia*-assigned ASVs indicated that the genus in this cohort comprised predominantly *A. muciniphila* (80.8% of *Akkermansia* reads) together with a substantial minority of *A. massiliensis* (16.7%), confirming that the genus-level signal largely, though not exclusively, reflects *A. muciniphila*. Nevertheless, the V3–V4 region could not distinguish *A. biwaensis* from “*Candidatus* A. timonensis”, and our data reflect relative (compositional) rather than absolute abundance; strain-level resolution and absolute quantification (e.g., using spike-in standards) therefore remain objectives for future work. Because *Akkermansia* occurred at very low abundance in many participants, low-level detections could be influenced by index (barcode) hopping; however, the diet–*Akkermansia* association was unchanged under a conservative 0.25% detection threshold, indicating that the finding was not driven by such low-abundance artifacts.

## 5. Conclusions

In this study, *Akkermansia* carriage was low among older Japanese adults, with the genus being undetectable in 45.3% of the participants and present at low abundance in the remainder. A noodle-based dietary pattern, but not rice, meat, or vegetables, was independently associated with lower *Akkermansia*; in a two-part analysis, this association was confined to whether *Akkermansia* was detected rather than to its abundance among carriers. However, the overall contribution of diet was small, the association was independent of measured dietary fiber, and no individual nutrient was significant; therefore, the underlying mechanism could not be elucidated. The presence or absence of *Akkermansia* was not meaningfully associated with grip strength, muscle mass, or frailty, and weak carrier-restricted associations did not survive correction for multiple testing. Collectively, these findings provide limited evidence for a role of *Akkermansia* in physical function among older Japanese adults and indicate that its low prevalence in this population warrants further, preferably longitudinal and mechanistic, investigation.

## Figures and Tables

**Table 1:** Adjusted association of PC4 (noodle-based dietary pattern) with *Akkermansia* CLR-Z; higher PC4 = greater noodle intake; *n* = 786.

Model	PC4 β	95% CI	*p*
Crude (PC4 only)	−0.143	−0.213 to −0.074	0.0001
Core (age, sex, BMI)	−0.121	−0.192 to −0.050	0.0009
Extended (+lifestyle/comorbidity)	−0.125	−0.197 to −0.053	0.0007
All 11 PCs + core	−0.117	−0.188 to −0.045	0.0014

**Table 2:** Two-part model of *Akkermansia* and physical function (adjusted for age, sex, BMI). Part 1: logistic (detection ~ phenotype); Part 2: linear (phenotype ~ *Akkermansia* CLR-Z) among carriers. FDR: Benjamini–Hochberg across nine physical-function models.

Two-Part Model	*n*	Estimate	95% CI	*p*	FDR-Adjusted *p*
Part 1—Grip (OR per 1 SD)	784	1.01	0.86 to 1.18	0.90	0.95
Part 1—Muscle (OR per 1 SD)	785	1.05	0.83 to 1.32	0.70	0.95
Part 1—Frailty (OR per 1 SD)	785	1.01	0.86 to 1.18	0.92	0.95
Part 2—Grip (β per 1 SD)	429	−0.198	−0.349 to −0.047	0.010	0.056
Part 2—Muscle (β per 1 SD)	430	−0.128	−0.228 to −0.028	0.013	0.056
Part 2—Frailty (β per 1 SD)	430	0.006	−0.146 to 0.157	0.94	0.95

## Data Availability

The raw 16S rRNA gene sequencing data generated in this study are openly available in the NCBI Sequence Read Archive under accession numbers PRJNA1470357 and PRJNA1440982. The subject-level identifier list (Microbiota-ID, age, sex, and BMI) is provided in Supplementary Table S2. Other individual-level cohort data are available from the corresponding author upon reasonable request owing to privacy restrictions.

## Supplementary Materials

The following supporting information can be downloaded at https://www.mdpi.com/article/10.3390/microorganisms14081729/s1, Figure S1. Food-group loading structure of the first five varimax-rotated principal components (PC1–PC5) of the 31 food groups (*n* = 786). Each panel shows the eight groups with the largest positive (blue) and the largest negative (red) rotated loadings. PC4 (fourth panel) is the component associated with *Akkermansia*: it loads strongly and positively on noodles and on salt/soy seasonings (which include noodle broth), with fried foods, pickles, and alcohol, and negatively on rice, green-and-yellow vegetables, and fruit. Because PC4 is inversely associated with *Akkermansia* abundance, a positive loading here corresponds to lower *Akkermansia*. Figure S2. Exploratory carrier-restricted associations of *Akkermansia* abundance with physical function (participants with detectable *Akkermansia* only; *n* ≈ 430). Scatterplots of centered-log-ratio (CLR)-transformed, Z-standardized *Akkermansia* abundance (x-axis) against Z-standardized grip strength, skeletal muscle mass, and frailty index. Red lines, ordinary least-squares fits; r, Pearson correlation. In covariate-adjusted models (age, sex, BMI), higher *Akkermansia* among carriers was weakly associated with lower grip strength (β = −0.198, *p* = 0.010) and muscle mass (β = −0.128, *p* = 0.013) and unrelated to frailty (*p* > 0.9). Because detection (presence vs absence) was unrelated to all three phenotypes (main text, Table 2) and abundances are very low, these associations are exploratory and are not emphasized in the main text. Figure S3. Exploratory diet-cluster analysis (hybrid Ward-to-k-means clustering of the 11 component scores; *n* = 786). Heatmap of mean standardized component scores (SD units) for the two data-driven clusters (C1, *n* = 484; C2, *n* = 302). The clustering was weak (silhouette ≈ 0.05), i.e., the data are better described as a continuum than as discrete groups. The clusters separated most strongly along PC8 (|C2 − C1| = 1.20), a Western-condiment-versus-traditional axis NOT associated with *Akkermansia* (β = −0.047, *p* = 0.19), and secondarily along PC4 (|C2 − C1| = 0.77), the noodle pattern associated with *Akkermansia*. Inference in the main text is therefore based on the continuous PC4 score rather than on the clusters. Figure S4. Spearman correlations of each of the 31 food groups (rows) with each varimax-rotated principal component (PC1–PC11; columns) in the full sample (*n* = 786). Colour denotes the correlation coefficient (red, positive; blue, negative); cells with |ρ| ≥ 0.30 are annotated; rows are ordered by their correlation with PC4, which is outlined. PC4 is dominated by noodles (ρ = 0.78) and salt/soy seasonings including noodle broth (ρ = 0.77), with smaller positive contributions from fried foods, pickles, and alcohol and negative contributions from rice, vegetables, and fruit; these noodle groups correlate strongly with PC4 but with no other component, showing that the noodle signal is specific to PC4. Other components capture distinct dietary axes (e.g., PC1, vegetables/seaweed/soy; PC2, fish; PC5, dairy and fruit). Figure S5. Genus-wide screen: The strongest genus-level associations with grip strength, skeletal muscle mass, and frailty (all 147 genera; linear regression adjusted for age, sex, and BMI; Benjamini–Hochberg FDR). Only Megasphaera was FDR-significantly (inversely) associated with grip strength, and no genus was FDR-significant for muscle mass or frailty. Table S1. Aggregation of the 70 BDHQ food items into 31 food groups. Table S2. Subject-level identifier list (Microbiota-ID, Subject-ID, sex, age, and BMI; *n* = 786); provided as a separate spreadsheet. Table S3. Associations of the 31 food groups with Akkermansia CLR-Z (adjusted for age, sex, BMI; Z-standardized; *n* = 786; sorted by *p*; Benjamini–Hochberg FDR). Table S4. Dietary fiber and the noodle–Akkermansia association (adjusted for age, sex, BMI; Z-standardized; *n* = 786). Across all 96 nutrients none reached FDR significance (min FDR *p* = 0.16). Table S5. Akkermansia abundance by exploratory diet cluster (Kruskal–Wallis *p* = 0.0011). Table S6. Screening of all 147 genus-level taxa for associations with Grip strength (linear regression adjusted for age, sex, and BMI; taxa as CLR-transformed z-scores; *n* = 786; Benjamini–Hochberg FDR). Table S7. Medication and comorbidity sensitivity analyses of the PC4-Akkermansia association (adjusted for age, sex, and BMI; *n* = 786). Table S8. Gastrointestinal, dietary, and social covariate sensitivity analyses of the PC4-Akkermansia association (progressive adjustment; *n* = 786). Table S9. Species-level assignment of Akkermansia amplicon sequence variants (V3–V4; ≥98% identity, unique best match). Table S10. Sensitivity of the PC4-Akkermansia detection association to the detection threshold (logistic regression adjusted for age, sex, and BMI; *n* = 786).
